# A research of STEAP1 regulated gastric cancer cell proliferation, migration and invasion in vitro and in vivos

**DOI:** 10.1111/jcmm.16038

**Published:** 2020-10-30

**Authors:** Zhe Zhang, Wen‐bin Hou, Chao Zhang, Yu‐en Tan, Dong‐dong Zhang, Wen An, Si‐wei Pan, Wan‐di Wu, Qing‐chuan Chen, Hui‐mian Xu

**Affiliations:** ^1^ Department of Oncology surgery The First Affiliated Hospital of China Medical University Shenyang China; ^2^ Department of Gastrointestinal surgery The First Affiliated Hospital of Sun Yat sen University Guangzhou China; ^3^ Department of Operation room The Second Affiliated Hospital of China Medical University Shenyang China

**Keywords:** gastric cancer, invasion, migration, proliferation, STEAP1

## Abstract

Six‐Transmembrane Epithelial Antigene of the Prostate 1 (STEAP1) is associated with the occurrence and development of cancer. This study aimed to clarify the role of STEAP1 in gastric cancer tumour growth and metastasis, as well as its molecular mechanism of action.Statistical methods were used for clinical data analysis. Protein expression was detected using immunohistochemistry(IHC). The mRNA and protein expression in the cell cultures were detected using reverse transcription‐polymerase chain reaction(RT‐PCR) and western blot analysis. Overexpression and silencing models were constructed using plasmid and lentivirus transfection. To detect cell proliferation in vitro, Cell Counting Kit‐8(CCK‐8), flow cytometry and colony formation assays were used; transwell and wound healing assays were used to detect cell migration and invasion;For in vivo experiments, nude BALB/c mice were used for detecting subcutaneous tumorigenesis and intraperitoneal implantation. In the results,we found STEAP1 was overexpressed in gastric cancer tissues and cell lines. Single‐factor and Cox analyses showed that STEAP1 gene expression level correlated with poor prognosis. Up‐regulation of STEAP1 increased cell proliferation, migration and invasion, which decreased after STEAP1 was knocked down. These changes were achieved via the activation of the AKT/FoxO1 pathway and epithelial‐mesenchymal transformation (EMT). The in vivo animal experiments showed that STEAP1 knock down, resulted in a decrease in the subcutaneous tumour and peritoneal tumour formation.

## 1 INTRODUCTION

Gastric cancer is a common kind of malignant tumour that seriously affects people's health.^1^ According to the data from the World health Organization (WHO), in contrast to the United States, Australia and New Zealand, China, Japan and Chile have higher incidence areas of gastric cancer.^2^ In China, gastric cancer is the second cause of cancer‐related deaths, with 679 000 new cases and 498 000 deaths.^3^ Gastric cancer is predisposed to occur in individuals aged between 50 and 70 years. However, in recent years, it has shown a ‘younger’ trend.^4^, ^5^, ^6^, ^7^, ^8^, ^9^ Therefore, it is essential to find new tumour markers to predict the risk of gastric cancer progression.

Tumour development involves many factors, which are controlled by many genes, including prostate transmembrane epithelial antigen. Six‐Transmembrane Epithelial Antigene of the Prostate (STEAP) was found as a prostate‐specific cell surface antigen using suppression subtractive hybridization technique for the first time.^10^, ^11^ STEAP is highly expressed in spontaneous transgenic mouse prostate cancer models and human prostate cancer. In addition, it is also expressed in the pancreas, ovary, gastrointestinal tract, cervix, testis, bladder, Ewing sarcoma and melanoma cells.^10^, ^12^ There are four members in the STEAP protein family, STEAP1‐4. The main focus of our study is STEAP1.

Gene STEAP1 is located in the 7q21.13 region of the human chromosome; it is 10.4 kb long and contains four introns and five exons. The transcription of the gene STEAP1 can produce two different kinds of mRNAs: a 1.4 kb and a 4 kb mRNAs. However, only the 1.4 kb mRNA can be processed into a mature protein, which contains 339 amino acids with molecular weight of 36 KD,^10^, ^12^ while the 4 kb mRNA contains a 2399 BP large intron, which is not translated into a mature protein.^13^ Data has shown that gene STEAP1 is closely related to communication between the adjacent cells, and it seemed to be beneficial for the occurrence and development of tumours.^14^ Its structural prediction and the location at the cell‐cell contacts indicated that gene STEAP1 product may be a transporter or channel.^10^, ^15^ Some previous research on different kinds of cancer have found that STEAP1 was observed in tumour tissue but not in normal tissue. The expression of STEAP1 was closely related to the malignant phenotype of cancer cells.^13^, ^16^, ^17^, ^18^, ^19^, ^20^ However, in Lee's study, they found no correlation between expression of STEAP1 and the clinicopathological factors.^21^ These conflicting results indicated that the roles of STEAP1 were varied depending on different cancer types. In our study, we will discuss the influence of STEAP1 on the proliferation, invasion and inflammatory reactions in gastric cancer.

## 2 MATERIALS AND METHODS

### 2.1 Clinical samples

212 samples of gastric cancer tissue and 60 samples of adjacent normal gastric tissue from patients were obtained from the First Affiliated Hospital of China Medical University from 2003 to 2010. None of the patients received preoperative chemotherapy or radiotherapy, and all of them were proven to have gastric cancer by pathology. The cancer tissue was fixed with formalin and preserved in paraffin. All pathological data were complete, and the postoperative follow‐up was sufficient. All patients were approved by the ethics committee of China Medical University to participate in the study and provided written informed consent.

### 2.2 Immunohistochemistry of human gastric cancer

To fix gastric cancer tissue samples, 10% formalin was used, then the tissue was paraffin‐embedded and cut into 4‐μm slices. The xylene and alcohol were used for dewaxing and rehydrating. Endogenous peroxidase activity was blocked by using hydrogen peroxide (30%), the citrate buffer (pH 6.0) was used to boil the sections fro 3 minutes in a pressure cooker. Next, normal goat serum was used for incubating the sections to reduce the nonspecific binding. Finally, the tissue sections were incubated (4°C, 12 hours) with anti‐STEAP1 antibody (1:200 dilution, B‐4, SC‐271872, Santa Cruz, USA). Enzyme labelled anti‐mouse/ rabbit IgG polymer was used for secondary antibody. (Mai Xin Biological company,Fuzhou,China). Finally, the sections were stained with diaminobezidin (DAB) for 60 seconds, stained with haematoxylin for 2 minutes and sealed with neutral resin. Fluorescence photographic microscope was used for obtaining images(Nikon,Japan).Two pathologists examined all tumour slides randomly. We evaluated STEAP1 staining intensity as follows: scored 0 (negative), 1 (weakly negative), 2 (weak positive) and 3 (strong positive). The percentage scores of positive cells per single field vision were as follows: scored 1 (0%‐25%), 2 (26%‐50%), 3 (51%‐75%) and 4 (76%‐100%). We multiplied the two scores above and obtained a final score ranging from 0 to 12. Tumour samples with a score < 6 were considered as negative expression; on the contrary, the score ≥ 6 was considered as positive expression.

### 2.3 Animals

Twenty‐four BALB/c nude female mice were purchased from Beijing Vital River Laboratory Animal Technology Co., Ltd. The mice were raised in the animal experimental centre of China Medical University. The twenty‐four nude mice were randomly divided into four groups. Two groups were used for hypodermic injection, and the other two groups were used for intraperitoneally injected.In the subcutaneous tumorigenesis experiment, 3 × 106 SGC‐7901 NC cells per mouse were injected in NC group, and 3*106 SGC‐7901 sh‐STEAP1 cells per mouse were injected in sh‐STEAP1 group.Similarly, in the experiment of intraperitoneal tumorigenesis, 3*106 SGC‐7901 NC cells per mouse were injected in NC group,and 3 × 106 SGC‐7901 sh‐STEAP1 cells per mouse were injected in sh‐STEAP1 group.The animals experiments primary antibodies were used as follows. STEAP1 (Ab207914,1:200) was purchased from Abcam. Ki‐67(GB111141,1:500), C‐CASPASE3(GB13436,1:200) were provided by Wuhan Servicebio technology Co., Ltd. IL1β(16806‐1‐AP, 1:200), IL6 (66146‐1‐AP, 1:200) were purchased from Proteintech Group, Wuhan, China. Secondary antibodies were used as follows, the goat anti‐rabbit (GB23303, 1:200) and anti‐mouse IgG (GB23301, 1:200) secondary antibodies were provided by Wuhan Servicebio technology Co., Ltd. All animal experimental steps were approved by the Animal Research Committee of China Medical University.

### 2.4 Cell culture

GES‐1, a normal gastric mucosa cell line was obtained from Nanjing Cobioer Biotechnology Co., Ltd. The gastric cancer cell lines MGC‐803 and SGC‐7901 were obtained from the Chinese Academy of Sciences. AGS was purchased from the American Type Culture Collection (ATCC, Manassas, VA, USA). MGC‐803, SGC‐7901 and GES‐1 cells were cultured in DMEM containing 10% FBS. AGS line was cultured in DMEM‐F12 medium containing 10% FBS. All cells were cultured at 37°C in a 5% CO2 incubator.

### 2.5 Cell transfection

ShRNA lentivirus was purchased from Shanghai Genechem Co., Ltd. The NC group insertion sequence was TTCTCCGAACGTGTCACGT. There were three shRNA sequences used (shRNA1: CCAACTTCATAATGGAACCAA; shRNA2: CAGCACACACAGGAACTCTTT; and shRNA3: AAGCTAGGAATTGTTTCCCTT). The STEAP1 containing cDNA plasmid and Flag empty plasmid were purchased from Beijing SinoBiological Co., Ltd. Lipofectamine 3000 reagent (Thermo Fisher Scientific, Inc) was used for plasmid transfection. The cells were harvested 48 hours after transfection. Western blot analysis and RT‐PCR were used to check the transfection efficiency.

### 2.6 RT‐PCR

TRIzol reagent was used to extracted total RNA. The reverse transcription kit PrimeScript RT was purchased from Takara. The primer designs were provided by Huada Gene Co., and the sequences were shown in Table 1.

**TABLE 1:** RNA primer sequence details

Genes	Forward/ Reverse	Sequences
GAPDH	Forward	5’‐GTCTCCTCTGACTTCAACAGCG‐3’
	Reverse	5’‐ACCACCCTGTTGCTGTAGCCAA‐3’
β‐actin	Forward	5’‐CACCATTGGCAATGAGCGGTTC‐3’
	Reverse	5’‐AGGTCTTTGCGGATGTCCACGT‐3’
CCND1	Forward	5’‐TCTACACCGACAACTCCATCC‐3’
	Reverse	5’‐TCTGGCATTTTGGAGAGGAAGTG‐3’
P27	Forward	5’‐ATAAGGAAGCGACCTGCAACCG‐3’
	Reverse	5’‐TTCTTGGGCGTCTGCTCCACAG‐3’
CDK4	Forward	5’‐CTCGTGCTGATGCTACTGAGGA‐3’
	Reverse	5’‐GGTCGGCGCAGTTGGGCTCC‐3’
E‐cadherin,	Forward	5’‐GCCTCCTGAAAAGAGAGTGGAAG‐3’
	Reverse	5’‐TGGCAGTGTCTCTCCAAATCCG‐3’
N‐cadherin	Forward	5’‐CCTCCAGAGTTTACTGCCATGAC‐3’
	Reverse	5’‐GTAGGATCTCCGCCACTGATTC‐3’
MMP‐2	Forward	5’‐AGCGAGTGGATGCCGCCTTTAA‐3’
	Reverse	5’‐CATTCCAGGCATCTGCGATGAG‐3’
MMP9	Forward	5’‐GCCACTACTGTGCCTTTGAGTC‐3’
	Reverse	5’‐CCCTCAGAGAATCGCCAGTACT‐3’
IL1β	Forward	5’‐CCACAGACCTTCCAGGAGAATG‐3’
	Reverse	5’‐GTGCAGTTCAGTGATCGTACAGG‐3’
IL6	Forward	5’‐AGACAGCCACTCACCTCTTCAG‐3’
	Reverse	5’‐TTCTGCCAGTGCCTCTTTGCTG‐3’

### 2.7 Western blot analysis

We used RIPA lysis buffer(purchased from Beyotime company) to lysis cells and obtain proteins. Proteins were separated using sodium dodecyl sulphate‐polyacrylamide gel electrophoresis (SDS‐PAGE, 8%). After transferring to a polyvinylidene fluoride (PVDF) membrane (Millipore, Billerica, MA, USA), the membranes were incubated overnight at 4°C with antibodies against STEAP1 (SC‐271872, 1:400) from Santa Cruz, USA. The other antibodies used are as follows. β‐actin (60008‐1‐Ig, 1:10 000)was purchased from Proteintech Group, Wuhan, China. P27 (3686, 1:1000), CDK4 (2546, 1:1000), AKT (4691, 1:1000), P‐AKT (4060, Ser473, 1:2000), FoxO1 (2880, 1:1000), P‐foxO1 (9464, 1:2000), E‐cadherin (14 472, 1:1000), N‐cadherin (13 116, 1:1000), Vimentin (5741, 1:1000), MMP‐2 (4022, 1:1000), MMP9 (13 667, 1:1000), were purchased from Cell Signaling Technology, USA. Then the goat anti‐rabbit and anti‐mouse IgG secondary antibodies were used to incubate the membrane at room temperature for 60 minute. Finally, the ECL was used to visualize and detect the proteins by using BioImaging Systems (UVP Inc, Upland, CA, USA).

### 2.8 Cell Counting Kit‐8 (CCK‐8) assay

SGC‐7901 and MGC‐803 cells were transfected with an empty vector, STEAP1 plasmid, negative control virus or sh‐STEAP1 virus. Cells were seeded at 3000 per well into a 96‐well plate, and CCK‐8 solution (Beyotime, Shanghai, China) was added into every well at 24, 48, 72 and 96 hours. Each group of cells was set with three auxiliary holes, repeat the experiment three times. A microplate reader was used to measure the absorbance values and estimate the cell proliferation rates.

### 2.9 Colony formation assay

For the colony formation assay, SGC‐7901 and MGC‐803 cells were transfected with plasmid or shRNA for 36 hours and plated into 6‐well cell plates (1000 cells/well). The cells were cultured in a 37°C incubator for 2‐3 weeks, fixed with alcohol for 30 minutes and stained with Trypan Blue for 20 minutes at room temperature. The colonies with more than 50 cells were counted. Finally, an HD camera was used to obtain the images.The experiment was repeated three times.

### 2.10 Flow cytometry

We used a flow cytometry assay to detect the cell cycle stage. SGC‐7901 and MGC‐803 cells were transfected with plasmid or shRNA and plated into 6‐well plates (1 × 10^5^ cells/well). After 24 hours, the cells were harvested using 0.25% trypsin in 1.5 mL Eppendorf tubes. Then, the cells were stained with propidium iodide (PI, 500 μ/tube, KeyGEN, Nanjing, China) at 37°C in the dark for 30 minutes. Finally, the cells were analysed using a FACSCalibur flow cytometer (Becton Dickinson, USA). The experiment was repeated three times.

### 2.11 Transwell assay

Cell migration experiments were performed using a 24‐well transwell chamber with a pore size of 8 μm (Costar). A total of 5 × 10^4^ cells in serum‐free DMEM were placed in the upper chamber, and DMEM with 10% FBS was added to the lower chamber. After more than 10 hours, the migration experiment was terminated, and the cells were observed in the medium below. Then, the cells on the membrane in the bottom chamber were fixed with 75% alcohol for 30 minutes and stained with Trypan Blue at room temperature for 20 minutes. Images were obtained using an inverted microscope. In addition, the transwell chamber was also used for cell invasion experiments. For these experiments, in addition to the above steps, Matrigel (1:9 dilution, BD Bioscience) was added to the upper chamber to observe the change in cell invasion ability.The experiment was repeated three times.

### 2.12 Wound healing assay

A wound healing assay was used to observe the migration of cells. In this study, 1 × 10^5^ cells were seeded into 6‐well plates for every group. After the cells had covered the entire plate, a pipette tip was used to make a scratch in the cell monolayer, and phosphate buffer saline (PBS) was used to wash the floating cells three times. Subsequently, we used serum‐free DMEM instead of the former medium. Finally, an inverted microscope (Olympus, Japan) was used to take images at 0 hour and 96 hours. The difference in scratch distance between the two phases can reflect the difference in the cell migration ability.The experiment was repeated three times.

### 2.13 Statistical analysis

GraphPad Prism 7.0 was used for image editing. SPSS 21.0 statistical software was used for data analysis.The data of three repeated experiments were input to analysis and expressed as the means ± SEMs. The chi‐square test was used to examine possible correlations between STEAP1 expression and clinicopathological factors. Survival rates were calculated using Kaplan‐Meier analysis. The log‐rank test was used for single‐factor analysis. Cox risk proportion model was used for multifactor analysis, and a value of *P* < .05 was considered statistically significant.

## 3 RESULTS

### 3.1 STEAP1 was highly expressed in gastric cancer tissue and closely connected with OS

**Figure 1:**
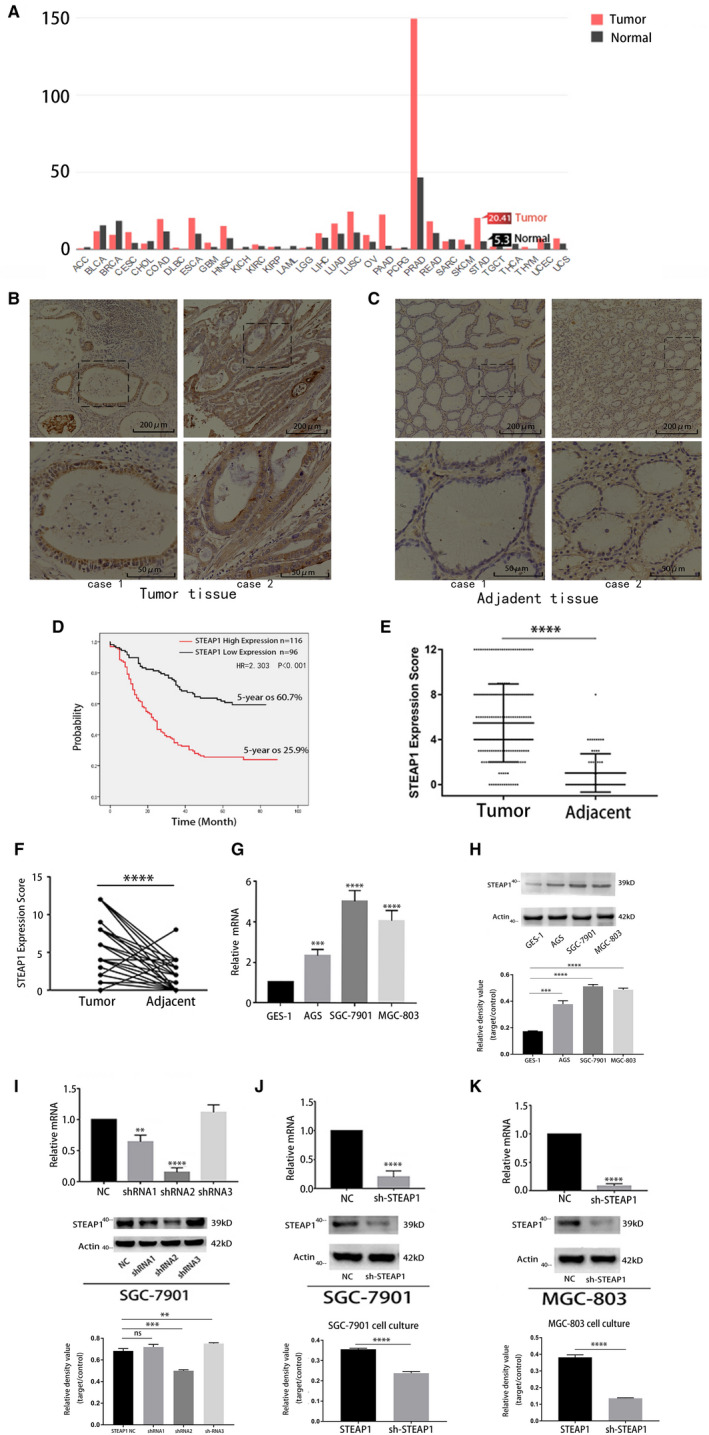
STEAP1 is highly expressed in gastric cancer and closely associated with OS; and STEAP1 lentivirus was successfully transfected into the cell line. (A) The GEPIA database shows the expression of STEAP1 in various types of cancer. (B) IHC assay shows the expression of STEAP1 protein in gastric cancer tissues (100x and 400x). (C) IHC assay shows the expression of STEAP1 protein in gastric paracancerous tissues (100x and 400x). (D) Kaplan‐Meier analysis was used to show the overall survival rates in gastric cancer patients with high and low expression levels of STEAP1. (E) The staining score distribution of 212 cases of gastric cancer and 60 cases of paracancerous tissues. (F) The staining score distribution of 60 matched tissues. (G) Detection of the expression of STEAP1 RNA expression levels in GES‐1, AGS, SGC‐7901 and MGC‐803 cells. (H) Detection of the protein expression levels of STEAP1 in GES‐1, AGS, SGC‐7901 and MGC‐803 cells. (I) Detection of the knockdown efficiency of three lentiviruses at the mRNA and protein levels using RT‐PCR and Western blot analysis, respectively. (J) Detection of STEAP1 knockdown efficiency in the SGC‐7901 cell line. (K) Detection of STEAP1 knockdown efficiency in the MGC‐803 cell line. OS, overall survival. (*P < .05, **P < .01, ***P < .001, ****P < .0001)

The GEPIA database showed that the STEAP1 gene was more highly expressed in gastric cancer than in the normal tissue (Figure 1A). We detected 212 cases of gastric cancer tissues and 60 cases of paracancerous tissues using IHC and scored them. The results showed that STEAP1 was highly expressed in cancer tissues and was mainly localized to the membrane and cytoplasm in cells (Figure 1B). The positive expression rate of STEAP1 was 54.7% (116/212). However, it was expressed at low levels or was negative in paracancerous tissues (Figure 1C). The 5‐year OS in the high expression group was 25.9%, which was significantly lower than 60.7% in patients with low expression group (*P < *.001, Fig. d). Figure 1E shows the detailed score in 212 cases of tumour tissues and in 60 cases of paracancerous tissues. The scores between the two groups were significantly different (*P* < .001). In addition, 60 matched tissues were scored, and the details are shown in Figure 1F (*P* < .001). The subsequent study of the data of 212 clinical cases showed that the factors that affected the prognosis in patients included tumour location (*P* = .029), tumour size (*P* = .012), Borrmann type (*P* = .019), STEAP1 expression (*P < *.001), N stage (*P* < .001), T stage (*P* < .001) and distant metastasis (*P* = .005) (Table 2). Cox multifactor analysis showed that the independent factors influencing the prognosis of patients included STEAP1 expression (*P* < .001), T stage (*P* = .005) and N stage (*P* < .001) (Table 3). In the study of the relationship between the expression of STEAP1 and clinicopathological factors, high expression of STEAP1 was closely related to Borrmann type (*P* = .009) and N stage (*P* < .001) (Table 4).

**TABLE 2:** Univariate analysis of prognostic factors in 212 patients

Clinicopathological factors	Patients (%) 212 (100))	5‐year OS (100%)	*P*‐value
Sex			0.637
Male	161(75.9)	42.9	
Female	51 (24.1)	39.2	
Age (years)			0.675
≤60	103(48.6)	42.7	
>60	109 (51.4)	41.3	
Location			0.029
Upper	26 (12.3)	38.5	
Middle	29(13.7)	44.8	
Lower	143 (67.5)	44.8	
Entire	14 (6.6)	14.3	
Size (cm)			0.012
≤4	55 (25.9)	56.4	
>4	157 (74.1)	36.9	
Borrmann Type			0.019
Borrmann 1	8(3.8)	50.0	
Borrmann 2	14 (6.6)	78.6	
Borrmann 3	185(87.3)	39.5	
Borrmann 4	5 (2.3)	20.0	
Differentiation degree^a^			0.472
Differentiated	102 (48.1)	39.2	
Undifferentiated	110 (51.9)	44.5	
STEAP1 expression status			0.000
Low	96 (45.3)	60.7	
High	116(54.7)	25.9	
Lymphovascular invasion			0.052
Negative	139(65.6)	61.5	
Positive	73(34.4)	32.9	
T staging			<0.001
T1‐2	33 (15.6)	57.6	
T3	34(16.0)	58.8	
T4a	139(65.6)	35.3	
T4b	6 (2.8)	16.7	
N staging			<0.001
N0	51(24.1)	70.6	
N1	48 (22.6)	45.8	
N2	35(16.5)	45.7	
N3a	50(23.6)	24.0	
N3b	28(13.2)	10.7	
M0 or M1			0.005
M0	201(94.8)	43.3	
M1	11(5.2)	10.7	

^a^ High and medium differentiated tubular adenocarcinoma and papillary adenocarcinoma were regarded as differentiated types; mucous adenocarcinoma, signet ring cell carcinoma, low and undifferentiated adenocarcinoma were regarded as undifferentiated types.

**TABLE 3:** Multifactorial analysis of prognostic factors in 212 patients

Clinicopathological factors	Exp(B)	95% CI	*P*‐value
Borrmann Type	0.934	0.783‐1.114	0.449
Location	1.131	0.700‐1.827	0.614
Size (cm)	0.919	0.573‐1.473	0.725
STEAP1 expression status	2.203	1.468‐3.300	<0.001
M0 or M1	1.329	0.652‐2.707	0.433
T staging	1.512	1.132‐2.020	0.005
N staging	1.343	1.162‐1.553	<0.001
Lymphovascular invasion	1.214	0.838‐1.759	0.304

**TABLE 4:** The relationship between clinicopathological factors and STEAP1 expression in 212 patients

Clinicopathological factors	Positive (cases)	Negative (cases)	*P*‐value
Sex			0.100
Male	78	83	
Female	18	33	
Age (years)			0.921
≤60	47	56	
>60	49	60	
Location			0.103
Upper	13	13	
Middle	14	15	
Lower	77	66	
Entire	12	2	
Size (cm)			0.510
≤4	27	28	
>4	69	88	
Borrmann Type			0.009
Borrmann 1	1	7	
Borrmann 2	4	10	
Borrmann 3	107	78	
Borrmann 4	4	1	
Differentiation degree^a^			0.823
Differentiated	47	55	
Undifferentiated	49	61	
Lymphovascular invasion			0.142
Negative	71	68	
Positive	45	28	
T staging			0.619
T1‐2	18	15	
T3	16	18	
T4a	60	79	
T4b	2	4	
N staging			<0.001
N0	15	36	
N1	27	21	
N2	19	16	
N3a	32	18	
N3b	23	5	
M0 or M1			0.542
M0	92	109	
M1	4	7	

^a^ High and medium differentiated tubular adenocarcinoma and papillary adenocarcinoma were regarded as differentiated types; mucous adenocarcinoma, signet ring cell carcinoma, low and undifferentiated adenocarcinoma were regarded as undifferentiated types.

### 3.2 Screening of the experimental cell lines and knockdown virus transfection

We detected the STEAP1 mRNA level in GES‐1, AGS, SGC‐7901 and MGC‐803 cells using RT‐PCR (Figure 1G). The results showed that STEAP1 was more highly expressed in SGC‐7901 and MGC‐803 cells. The western blot analysis yielded the same conclusion (Figure 1H). Therefore, we selected SGC‐7901 and MGC‐803 cells as experimental cell lines. We overexpressed and knocked down the STEAP1 gene by transfecting the STEAP1 plasmid and STEAP1 shRNA. We transfected negative control (NC) virus and three kinds of STEAP1‐shRNAs into SGC‐7901 cells and detected the knockdown efficiency on mRNA and protein levels using RT‐PCR and western blot analysis. The results showed that shRNA2 had the highest knockdown efficiency (Figure 1I). Subsequently, we transfected NC virus and STEAP1‐shRNA2 into SGC‐7901 and MGC‐803 cells. The results showed successful transfection and knockdown using RT‐PCR and western blot analysis (Figure 1J‐k).

### 3.3 STEAP1 gene regulates the cell cycle via the Akt/FoxO1 pathway to influence cell proliferation

**Figure 2:**
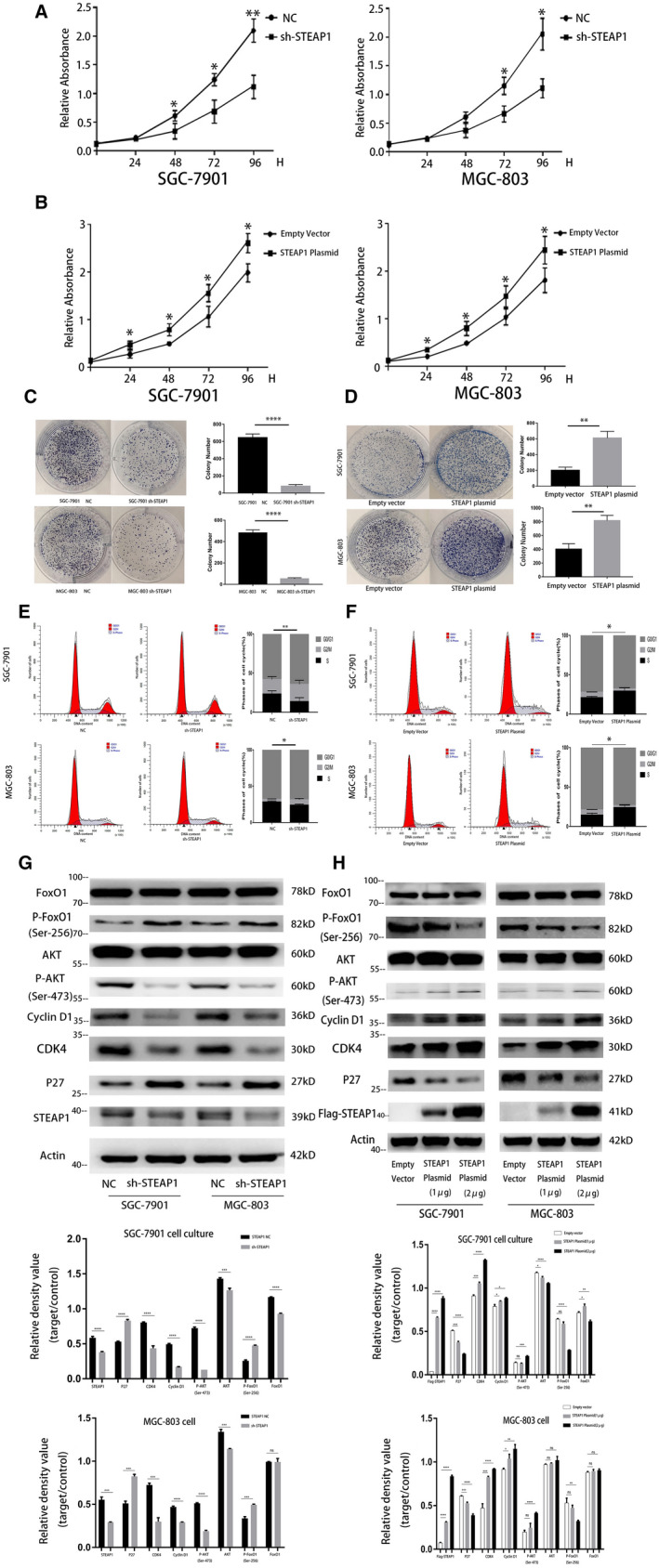
Functional experiments related to cell proliferation in the SGC‐7901 and MGC‐803 cell lines. (A) CCK‐8 assay showed the differences in cell proliferation before and after knocking down STEAP1 in the SGC‐7901 and MGC‐803 cell lines. (B) CCK‐8 assay showed the differences in cell proliferation before and after overexpression of STEAP1 in the SGC‐7901 and MGC‐803 cell lines. (C) Colony formation assay showed the differences in cell proliferation before and after knocking down STEAP1 in the SGC‐7901 and MGC‐803 cell lines. (D) Colony formation assays showed the differences in cell proliferation before and after STEAP1 overexpression in the SGC‐7901 and MGC‐803 cell lines. (E) Flow cytometry detected the differences in cell cycle before and after knocking down STEAP1 in the SGC‐7901 and MGC‐803 cell lines. (F) Flow cytometry detected the difference in cell cycle before and after the overexpression of STEAP1 in the SGC‐7901 and MGC‐803 cell lines. (G) Western blot analysis detected the differences in protein expression related to the cell cycle and pathway before and after knocking down STEAP1 in the SGC‐7901 and MGC‐803 cell lines. (H) Western blot analysis detected the differences in protein expression related to the cell cycle and pathway before and after the overexpression of STEAP1 in the SGC‐7901 and MGC‐803 cell lines. (*P < .05, **P < .01, ***P < .001, ****P < .0001)

The CCK‐8 assay results showed that the absorbance in the sh‐STEAP1 group was lower than that in the NC group at 48, 72 and 96 hours in both the SGC‐7901 and MGC‐803 cell lines (Figure 2A). In another group of comparisons, we found that the absorbance of the STEAP1 plasmid vector group was higher than that of the NC group at 24, 48, 72 and 96 hours in both the SGC‐7901 and MGC‐803 cell lines (Figure 2B). The CCK‐8 results indicated that the STEAP1 gene can influence cell proliferation. In the colony formation assay, we found that when STEAP1 was knocked down, the colony number was lower than that in the NC group (Figure 2C), while when STEAP1 was overexpressed, the colony number was higher than that in the empty vector group (Figure 2D). The colony formation assay results also indicated that the STEAP1 gene can influence cell proliferation. Next, we used flow cytometry to detect the cell cycle. The results showed that when STEAP1 was knocked down, the percentage of the cells in S phase was decreased, and the percentages of cells in G0/G1 and G2/M phase was increased in both SGC‐7901 and MGC‐803 cells (Figure 2E). When STEAP1 was overexpressed, the percentage of S phase cells was increased, and the percentages of G0/G1 and G2/M phase cells was decreased in both SGC‐7901 and MGC‐803 cells (Figure 2F). The flow cytometry assay indicated that the STEAP1 gene can influence cell proliferation by influencing the cell cycle. Finally, we detected cell cycle related proteins and pathway proteins to identify the underlying mechanism. The Western blot analysis showed that when STEAP1 was down regulated, CDK4,Cyclin D1,total AKT (AKT) and phosphorylated AKT (P‐AKT) were down regulated, P27 and phosphorylated FoxO1 (P‐FoxO1) were up‐regulated both in SGC‐7901 and MGC‐803. In addition, we found total FoxO1 (FoxO1) was down regulated in SGC‐7901 while had no significant change in MGC‐803 (Figure 2G). When STEAP1 was up‐regulated, CDK4 and Cyclin D1 were relatively up‐regulated, and P27 was down regulated. AKT and FoxO1 almost showed no significant change, while P‐AKT was up‐regulated and P‐FoxO1 was down regulated both in SGC‐7901 and MGC‐803(Figure 2H).

### 3.4 STEAP1 regulates cell migration and invasion via EMT

**Figure 3:**
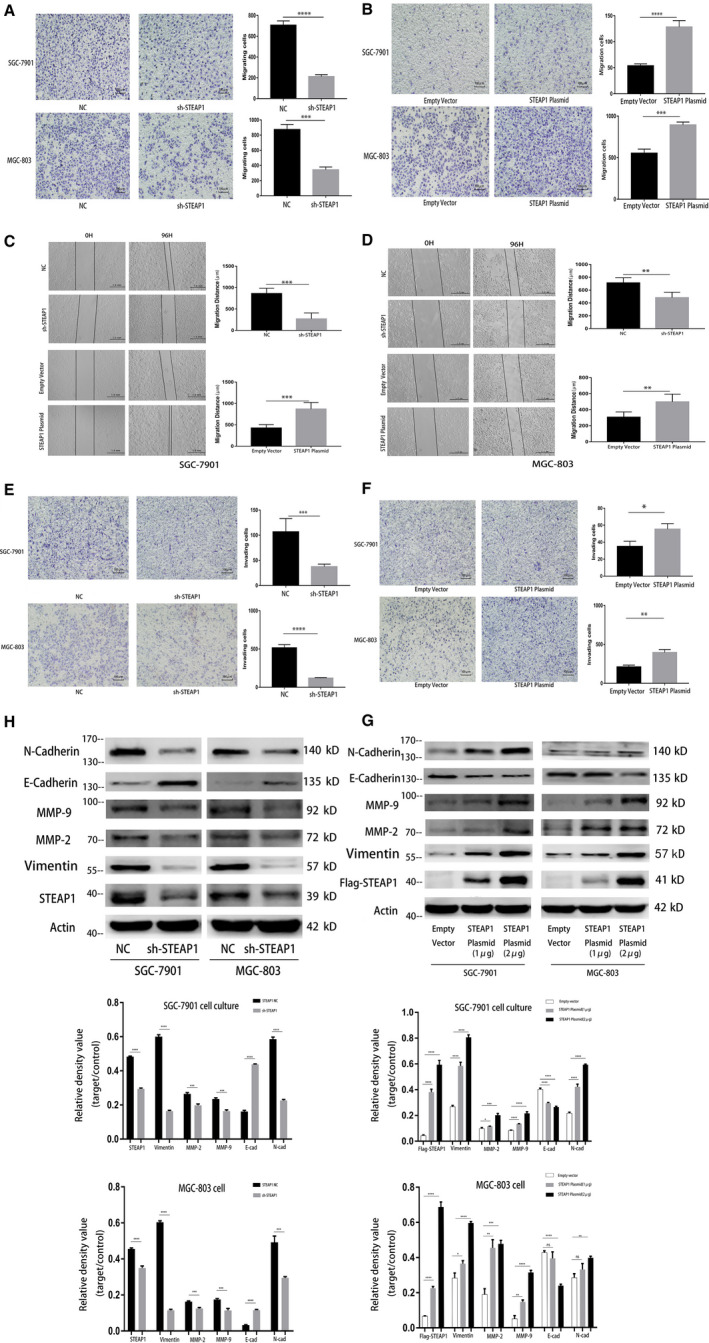
Functional experiments related to cell migration and invasion in the SGC‐7901 and MGC‐803 cell lines. (A) Detection of cell migration differences before and after knocking down STEAP1 in the SGC‐7901 and MGC‐803 cell lines using transwell assay. (B) Detection of cell migration differences before and after the overexpression of STEAP1 in the SGC‐7901 and MGC‐803 cell lines by transwell assay. (C) Detection of cell migration differences before and after knocking down STEAP1 in the SGC‐7901 and MGC‐803 cell lines using wound healing assay. (D) Detection of cell migration differences before and after the overexpression of STEAP1 in the SGC‐7901 and MGC‐803 cell lines using wound healing assay. (E) Detection of cell invasion differences before and after knocking down STEAP1 in the SGC‐7901 and MGC‐803 cell lines using transwell assay. (F) Detection of cell invasion differences before and after the overexpression of STEAP1 in the SGC‐7901 and MGC‐803 cell lines using transwell assay. (G) Western blot analysis detected the difference in protein expression related to cell migration, invasion and EMT before and after knocking down STEAP1 in the SGC‐7901 and MGC‐803 cell lines. (H) Western blot analysis detected the difference in protein expression related to cell migration, invasion and EMT before and after the overexpression of STEAP1 in the SGC‐7901 and MGC‐803 cell lines. (*P < .05, **P < .01, ***P < .001, ****P < .0001)

The transwell assay results showed that the number of migrating cells in the sh‐STEAP1 group was lower than that in the NC group in both SGC‐7901 and MGC‐803 cells (Figure 3A). The number of migrating cells in the STEAP1 plasmid group was higher than that in the empty vector group (Figure 3B). The results of the wound healing assay also provided consistent conclusions. In SGC‐7901 cells, the migration distance between 0 and 96 hours in the NC group was more obvious than that in the sh‐STEAP1 group. The migration distance of the empty vector group was shorter than that of the STEAP1 plasmid group (Figure 3C). This result was also identified in MGC‐803 cells. The migration distance of the NC group was longer than that of the sh‐STEAP1 group, and the migration distance of the empty vector group was shorter than that of the STEAP1 plasmid group (Figure 3D). The results of the above two experiments indicated that when STEAP1 was knocked down, the cell migration ability was decreased, whereas it increased after the overexpression of STEAP1. We also used a transwell assay and placed Matrigel into the upper chamber for detecting the effect of the STEAP1 gene on cell invasion. The results showed that the number of invading cells in the sh‐STEAP1 group was lower than that in the NC group in both SGC‐7901 and MGC‐803 cells (Figure 3E). The number of invading cells in the STEAP1 plasmid group was higher than that in the empty vector group (Figure 3F). These results indicated that the STEAP1 gene had an effect on cell invasion. When STEAP1 was knocked down, the cell invasion ability was decreased and increased after overexpressing STEAP1. Finally, we detected cell migration‐ and invasion‐related proteins using western blot analysis. The results showed that Vimentin, N‐cadherin, MMP‐2 and MMP‐9 were down‐regulated and E‐cadherin was up‐regulated after we down‐regulated the gene STEAP1 (Figure 3G). In contrast, when STEAP1 was overexpressed, Vimentin, N‐cadherin, MMP‐2 and MMP‐9 were relatively up‐regulated, and E‐cadherin was down‐regulated (Figure 3H).

### 3.5 In vivo animal experiments

**Figure 4:**
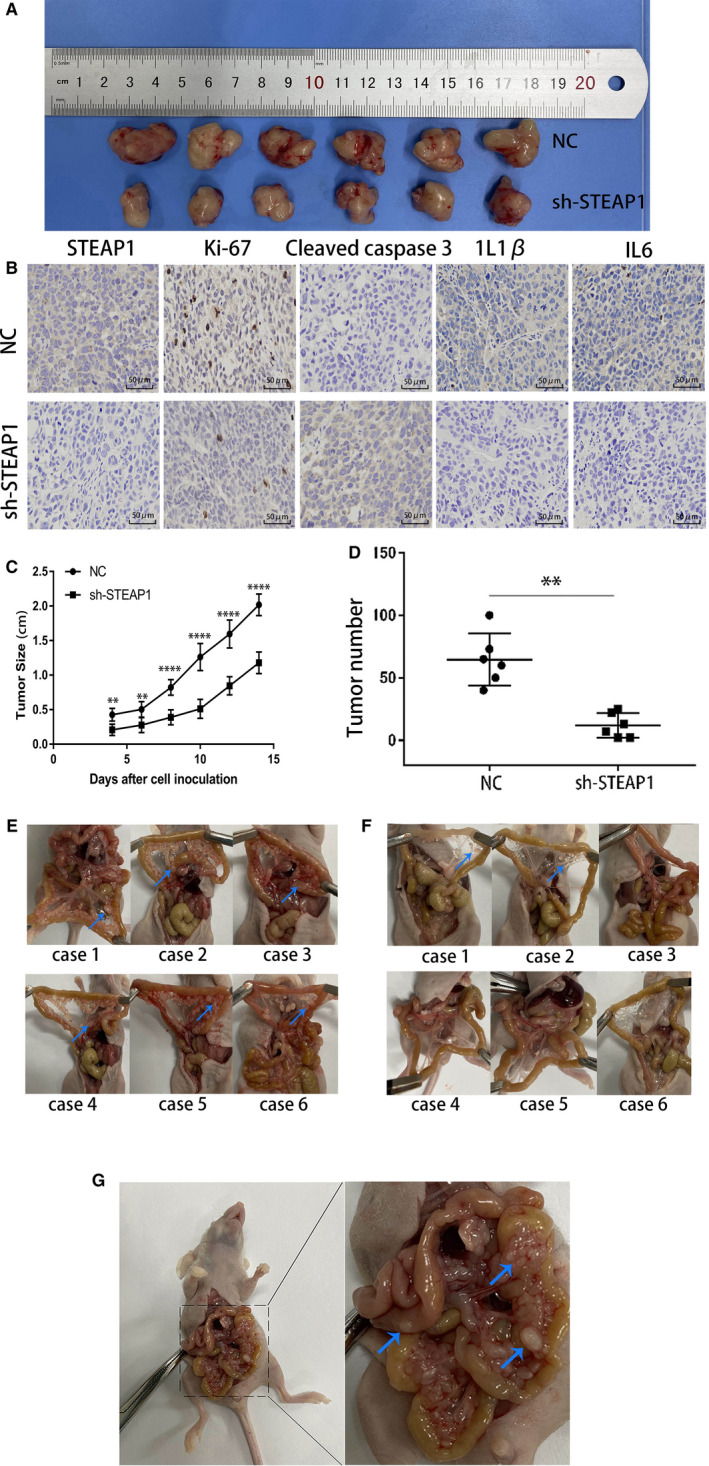
This figure shows the effect of the STEAP1 gene on cell proliferation and metastasis invivo through animal experiments. (A) The tumorigenesis experiment in nude mice shows the tumour size differences between the SGC‐7901 NC group and the sh‐STEAP1 group. (B) IHC assayshows the expression of cell proliferation‐ and inflammation‐related proteins in the tumours of nudemice (400x). (C) The difference in tumour size between the SGC‐7901 NC group and sh‐STEAP1 group in two weeks. (D) The tumour number difference between the SGC‐7901 NC group and sh‐STEAP1 group. (E) The number and distribution of abdominal tumours in 6 mice of NC group were shown. The location of mesenteric tumours was marked by the arrows (F) The number and distribution of abdominal tumours in 6 mice of sh‐STEAP1 group were shown. The location of mesenteric tumours was marked by the arrows (G) The tumours were found on the mesentery and the wall of the intestine, several larger tumours on the mesenteric and intestinal wall were marked by the arrows..(*P < .05, **P < .01, ***P < .001, ****P < .0001)

Twelve BALB/c nude mice were used to study tumour formation and were randomly divided into two groups. We subcutaneously injected 3 × 10^6^ of SGC‐7901 NC cells per mouse in the first group, which was called the NC group. The mice in the other group were subcutaneously injected with 3 × 10^6^ of SGC‐7901 and sh‐STEAP1 cells per mouse, which was called sh‐STEAP1 group. The tumour sizes were measured every 2 days from 4 to 14 days after the injection and the results are shown in Figure 4C. Twelve tumour specimens were removed from the mice on the 14th day. The results showed that the tumour size in the NC group was larger than that in the sh‐STEAP1 group (Figure 4A). Then, we carried out paraffin embedding, sectioning and IHC experiments with the tumour tissue. The results showed that the expression of Ki67, IL‐1β and IL‐6 in the NC group was higher than that in the sh‐STEAP1 group, while cleaved caspase‐3 expression was lower than that in the sh‐STEAP1 group (Figure 4B). In the intraperitoneal tumorigenesis experiment, similar to the subcutaneous tumorigenesis experiment, twelve mice were divided into the NC and sh‐STEAP1 groups, and 3 × 106 cells per mouse were injected by intraperitoneal injection. Three weeks later, the mice were sacrificed to observe the number of intraperitoneal tumours, including the mesentery, on the wall of the intestine.The number of tumours in the abdominal cavity of the two groups were statistically analysed and the difference was statistically significant (Figure 4D). Figure 4E showed the general distribution of tumours of NC group..We used haemostatic forceps to clamp the two sides of the intestine to expand the mesentery, we found a huge number of tumours in the mesentery arranged like beads (Figure 4E.). However, tumours were rare or absent in the sh‐STEAP1 group (Figure 4F). We enlarged the image of one case in NC group when the abdominal cavity of nude mice was just opened. We can clearly see that the tumours covered the abdominal cavity, and several larger tumours on the mesenteric and intestinal wall were marked at the arrow. (Figure 4G).

## 4 DISCUSSION

As a global health problem, cancer affects the quality of patients’ lives worldwide and causes thousands of deaths every year.^1^ STEAP1 is overexpressed in many kinds of cancers, such as prostate cancer, colon cancer, bladder cancer, ovarian cancer, pancreatic cancer, testicular cancer, breast cancer, cervical cancer and Ewing sarcoma.^10^, ^12^ In our study, STEAP1 was overexpressed in gastric cancer and closely related to the prognosis of patients (Figure 1B). The 5‐year OS of patients with a low expression of STEAP1 was 60.7%, while that of patients with a high expression of STEAP1 was only 25.9% (Figure 1D). STEAP1 plays a role as an oncogene in gastric cancer, and this result was consistent with the conclusion that STEAP1 is an oncogene in other kinds of cancer. Tumour cell growth, metastasis, proliferation, migration and invasion are basic biological functions.^22^ We down‐regulated or up‐regulated STEAP1 by using lentivirus knockdown or STEAP1 plasmids, respectively, to detect the changes in the above functions. The results of the CCK‐8 and colony formation assays indicated that when we overexpressed STEAP1, the percentage of cells in the S phase increased, that in the G0/G1 and G2/M phases decreased, and the cell proliferation ability was also improved. In contrast, when STEAP1 was knocked down, the percentage of cells in the S phase decreased, that in the G0/G1 and G2/M phases increased, and cell proliferation was reduced (Figure 2A‐F). Cyclin D1 is known as an oncogene and overexpressed in many kinds of cancers.^23^ By binding with CDK4 ( a partner kinases of cyclin D1), cyclin D1 can release transcription factors and advance cell cycle progression from the G1 phase to the S phase. The P27 protein limits cell cycle progression, mainly by inhibiting complex formation, such as CyclinD1‐CDK4 and CyclinE‐CDK2, to block the cell cycle in the G1 phase. Previous studies have found that the AKT pathway is one of the main signalling pathways influencing cancer cell proliferation.^24^, ^25^, ^26^, ^27^, ^28^, ^29^ Therefore, it was reasonable to consider that STEAP1 can affect cell proliferation via the AKT pathway. Our results also showed that when STEAP1 was down‐regulated, P‐AKT, CDK4 and Cyclin D1 were relatively down‐regulated, and P27 and P‐FoxO1 were up‐regulated (Figure 2G). When STEAP1 was up‐regulated, P‐AKT, CDK4 and Cyclin D1 were relatively up‐regulated, and P‐FoxO1 and P27 were down‐regulated. P‐AKT was up‐regulated and P‐FoxO1 was down‐regulated (Figure 2H). These results indicated that STEAP1 can regulate the cell cycle via the Akt/FoxO1 pathway to influence cell proliferation. The results of the transwell and wound healing assays showed that when we overexpressed STEAP1, cell migration and invasion increased. In contrast, when STEAP1 was knocked down, the two abilities above decreased (Figure 3A‐F). Next, EMT‐related proteins, MMP2 and MMP9 were detected by western blotting. The EMT‐related proteins include N‐cadherin,vimentin and E‐cadherin. A previous study showed that when cells tend to migrate and metastasize, the protein expression of vimentin and N‐cadherin increases and that of E‐cadherin decreases.^30^, ^31^ In addition, many studies have identified that MMP2 and MMP9 are closely related to tumour migration and invasion and explained the mechanism.^32^, ^33^, ^34^ The results of our study showed that when STEAP1 was overexpressed, N‐cadherin, Vimentin, MMP‐2 and MMP‐9 were relatively up‐regulated, and E‐cadherin was down‐regulated. When STEAP1 was down‐regulated, N‐cadherin, MMP‐9 and MMP‐2 were down‐regulated, and E‐cadherin was up‐regulated (Figure 3H‐g). These results indicated that the STEAP1 gene.

may regulate cell migration and invasion via EMT. The result of the apoptosis marker cleaved caspase‐3 and proliferation‐related nuclear antigen Ki‐67 by IHC assay also indicated that STEAP1 plays a very important role in cell proliferation in vitro. Until the early 1990s, We always admitted that peritoneal metastasis of gastric cancer is a kind of terminal disease and the effect of systemic chemotherapy is limited for it. In our study, we established a peritoneal metastasis model by intraperitoneal injection of tumour cells into mice. The results showed that the number of tumours on the mesentery in the NC group was higher than that in the sh‐STEAP1 group. Through this experiment, we verified the effect of STEAP1 on tumour cell invasion and metastasis in vivo.

### 4.1 Conclusions

In conclusion, STEAP1 was overexpressed in gastric cancer and closely connected with OS. STEAP1 can regulate the cell cycle via the Akt/FoxO1 pathway to influence cell proliferation. STEAP1 may affect cell migration and invasion via EMT induction.

## CONFLICTS OF INTEREST

There is no competing interests.

## AUTHOR CONTRIBUTION

**Zhe Zhang:** Conceptualization (equal); Writing‐original draft (lead). **Wen bin Hou:** Writing‐review & editing (equal). **Chao Zhang:** Methodology (equal). **Yu en Tan:** Investigation (equal). **Dong dong Zhang:** Validation (equal). **Wen An:** Supervision (equal). **Si wei Pan:** Data curation (equal). **Wan di Wu:** Writing‐original draft (equal). **Qing chuan Chen:** Software (equal). **huimian Xu:** Funding acquisition (lead); Project administration (lead); Resources (lead).
