# CORRIGENDUM

**DOI:** 10.1111/jcmm.17117

**Published:** 2022-01-08

In Zhe Zhang et al,^1^ the image for STEAP1 Plasmid/SGC‐7901 in Figure 3F overlapped with Figure 3E due to technical error during image preparation. The correct figure is shown below. The authors confirm all results and conclusions of this article remain unchanged.

**FIGURE 3:**
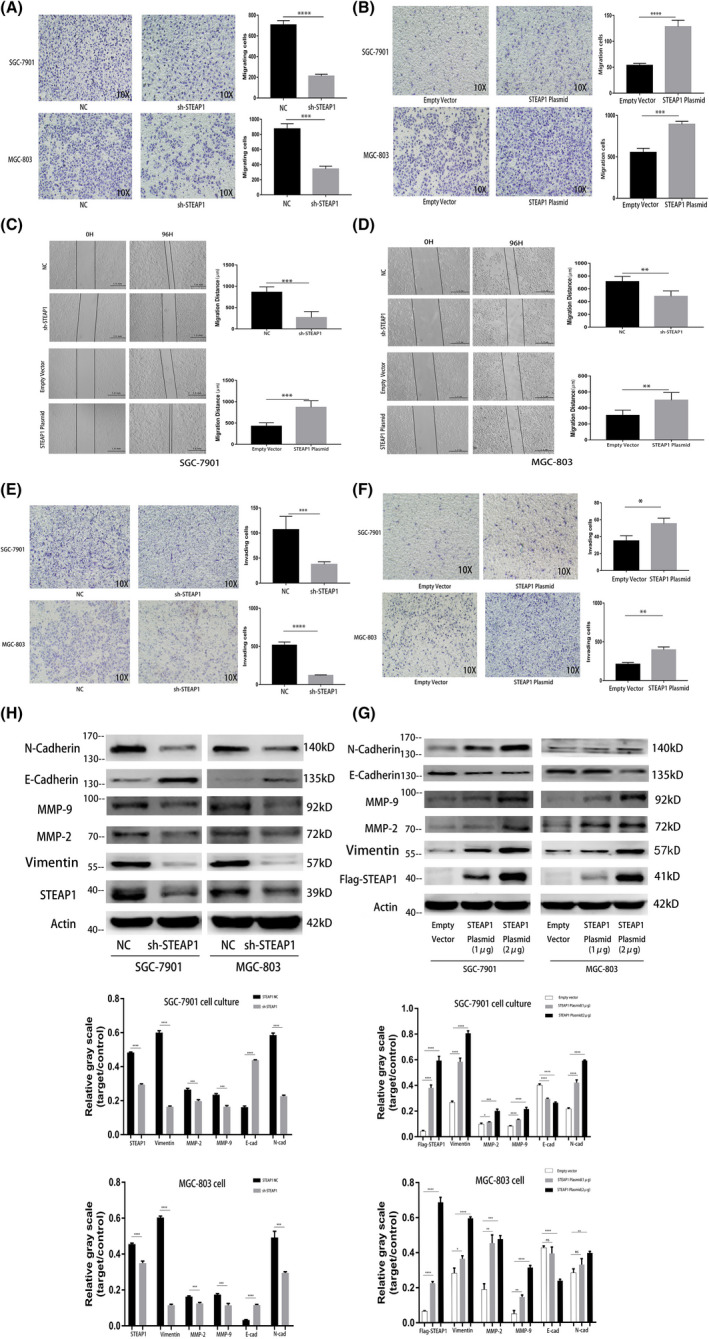
Functional experiments related to cell migration and invasion in the SGC‐7901 and MGC‐803 cell lines. (A) Detection of cell migration differences before and after knocking down STEAP1 in the SGC‐7901 and MGC‐803 cell lines using transwell assay. (B) Detection of cell migration differences before and after the overexpression of STEAP1 in the SGC‐7901 and MGC‐803 cell lines by transwell assay. (C) Detection of cell migration differences before and after knocking down STEAP1 in the SGC‐7901 and MGC‐803 cell lines using wound healing assay. (D) Detection of cell migration differences before and after the overexpression of STEAP1 in the SGC‐7901 and MGC‐803 cell lines using wound healing assay. (E) Detection of cell invasion differences before and after knocking down STEAP1 in the SGC‐7901 and MGC‐803 cell lines using transwell assay. (F) Detection of cell invasion differences before and after the overexpression of STEAP1 in the SGC‐7901 and MGC‐803 cell lines using transwell assay. (G) Western blot analysis detected the difference in protein expression related to cell migration, invasion and EMT before and after knocking down STEAP1 in the SGC‐7901 and MGC‐803 cell lines. (H) Western blot analysis detected the difference in protein expression related to cell migration, invasion and EMT before and after the overexpression of STEAP1 in the SGC‐7901 and MGC‐803 cell lines (*p < 0.05, **p < 0.01, ***p < 0.001, ****p < 0.0001)
